# Outcome measures of instrumented gait analysis in hereditary spastic paraplegia: a systematic review

**DOI:** 10.1186/s12984-025-01646-4

**Published:** 2025-06-05

**Authors:** Veronika Koch, Alzhraa Ibrahim, Juergen Winkler, Bjoern Eskofier, Martin Regensburger, Heiko Gassner

**Affiliations:** 1 Fraunhofer Institute for Integrated Circuits IIS, Erlangen, Germany; 2 Machine Learning and Data Analytics Lab, Department Artificial Intelligence in Biomedical, Engineering (AIBE), Friedrich-Alexander-Universität Erlangen-Nürnberg (FAU), Erlangen, Germany; 3 Department of Molecular Neurology, University Hospital Erlangen, Friedrich-Alexander-Universität, Erlangen-Nürnberg (FAU), Erlangen, Germany; 4 Center for Rare Diseases Erlangen (ZSEER), University Hospital Erlangen, Erlangen, Germany; 5 Translational Digital Health Group, Institute of AI for Health, German Research Center for Environmental Health, Helmholtz Zentrum München, Neuherberg, Germany

**Keywords:** Hereditary spastic paraplegias, Motion capture, Gait analysis, Sensors, Mobile sensor systems, Inertial measurement unit

## Abstract

**Background:**

Hereditary spastic paraplegias (HSPs) comprise a group of genetic movement disorders characterized by progressive spasticity and weakness of the lower limbs leading to gait deficits. Instrumented gait measures are applied to quantify gait patterns in HSP objectively. However, there is no consensus on the most relevant HSP-specific digital outcome measures for future clinical studies.

**Aim:**

This systematic review aims to summarize outcome measures of instrumented gait analysis in HSP patients, focusing on both traditional motion capture (MOCAP) and inertial sensor systems.

**Methods:**

Following PRISMA-2020 guidelines, a comprehensive literature search was conducted in PubMed, Scopus, and Web of Science to identify studies using instrumented gait analysis in HSP. Data on participant characteristics, measurement systems, outcome measures, results, and risk of bias were systematically extracted.

**Results:**

In total, 38 studies published between 2004 and 2024, including 29 observational studies and 9 interventional studies, met the inclusion criteria. Various gait parameters were used, including spatio-temporal, kinematic, kinetic, and electromyography (EMG) parameters. Walking speed and range-of-motion (ROM) knee were identified as important parameters for differentiating HSP patients from healthy controls, but these parameters are more general rather than disease-specific. Foot lift, ROM foot, and gait variability are promising, more disease-specific parameters, as they reflect disease severity and increased balance deficits. However, a deeper understanding of all gait parameter categories is necessary, particularly for the upper body. Few studies explored sub-cohorts that exhibit different HSP gait characteristics.

**Conclusion:**

While MOCAP provides valuable data in controlled hospital environments, there is a need for validated mobile sensor systems capturing the gait patterns of HSP patients in real-life without supervision. Future research must focus on better longitudinal multicenter studies with larger sample sizes to establish robust digital outcomes and monitor disease progression and therapeutic response in HSP.

**Supplementary Information:**

The online version contains supplementary material available at 10.1186/s12984-025-01646-4.

## Introduction

Hereditary spastic paraplegias (HSPs) are a heterogeneous group of rare genetic movement disorders with less than 10 cases per 100.000 individuals [1]. They are mainly characterized by progressive spasticity and weakness of the lower limbs, often resulting in progressively aggravating gait deficits [2, 3]. HSPs are clinically divided into two forms: a pure and a complicated form. The complicated HSPs are characterized by additional neurological or non-neurological symptoms (e.g., dementia, muscle atrophy, ataxia) [1, 4]. More than 90 genetic types of HSP have been described [1]. The age of onset varies from infancy to late adulthood with a balanced gender distribution. To date, treatment of HSP is limited to symptomatic treatment, including medications (e.g., botulinum toxin injections), orthotics (e.g., ankle–foot orthoses or electrical stimulation), and physical therapy [1].

The most commonly used scale to measure disease severity and progression is the Spastic Paraplegia Rating Scale (SPRS) [5, 6]. Functional gait tests (e.g., 10 m walking test, 2 min walk test) [6] provide semi-quantitative outcome measures to assess gait functions. They are inexpensive and easy-to-apply. The outcome measures of functional gait tests highly correlate with the SPRS score [7] since the 10 m walking test and 2 min walk test evaluate walking speed and endurance, respectively. As the SPRS primarily assesses ‘Walking distance without pause’, ‘Gait quality’, ‘Maximum gait speed’, and ‘Climbing stairs’, functional gait tests do not provide added value. In particular, distinct features, including subtle gait deficits not observable by clinical raters, cannot be detected [8–10].

Using instrumented gait analysis, HSP-specific gait patterns and subtle gait deficits may be objectively quantified. The gold standard in instrumented gait analysis is three-dimensional motion capture (MOCAP). These systems are highly accurate and precise but expensive, require a time-consuming setup and data recording process [11], and are challenging to integrate into clinical routine examinations [12]. In contrast, there are instrumented gait analysis systems using inertial sensors or instrumented mats, which are mobile, easy-to-apply, and provide results after a short period. Recently, validated inertial sensor systems have gained importance for gait analysis as an alternative to MOCAP, as they also provide the ability to collect real-life mobility data [11]. Additionally, instrumented gait analysis may be used to discriminate between HSP and cerebral palsy (CP) [13] and to quantify gait changes in prodromal HSP gene carriers [14]. For neurodegenerative diseases such as HSP, electromyography (EMG) combined with kinematic, kinetic, and spatio-temporal parameters is an essential measure of instrumented gait analysis [15] as it objectively detects muscle stiffness and spasticity, which is of major importance for interpreting gait deficits.

However, there is no structured overview available that focuses on digital outcome measures of instrumented gait analysis in HSP patients. Existing reviews on instrumented gait analysis did not include inertial sensor systems [16]. Four reviews focus on different treatment methods for HSP patients and include results from gait analysis as an outcome measure [6, 17–19]. The latest review focused on general outcome measures and biomarkers for HSP [6]. Therefore, this systematic review aimed to summarize the outcome measures of instrumented gait analysis ranging from inertial sensors to different MOCAP systems and identify the most important HSP-specific digital outcomes based on the existing literature.

## Methods

The systematic review was planned, conducted, and reported according to the PRISMA-2020 statement guidelines for reporting systematic reviews [20].

### Eligibility criteria and search strategy

A literature search was performed in the 3 databases: PubMed, Scopus, and Web of Science in March 2025, including all articles published until March 24, 2025, without defining a starting date. Solely English-language and original research articles were included. Conference abstracts, review articles, data articles, commentaries, grey literature, and study protocols were excluded. In addition, reference lists of included and relevant review articles were searched.

The search string was developed based on the PICO framework and was used for each database. The PICO framework included:

- Population: patients with pure or complicated HSP
- Intervention: detect gait patterns using instrumented gait analysis
- Comparison: healthy controls or no intervention group
- Outcome: gait parameters or other outcome measures of instrumented gait analysis

**Fig. 1:**
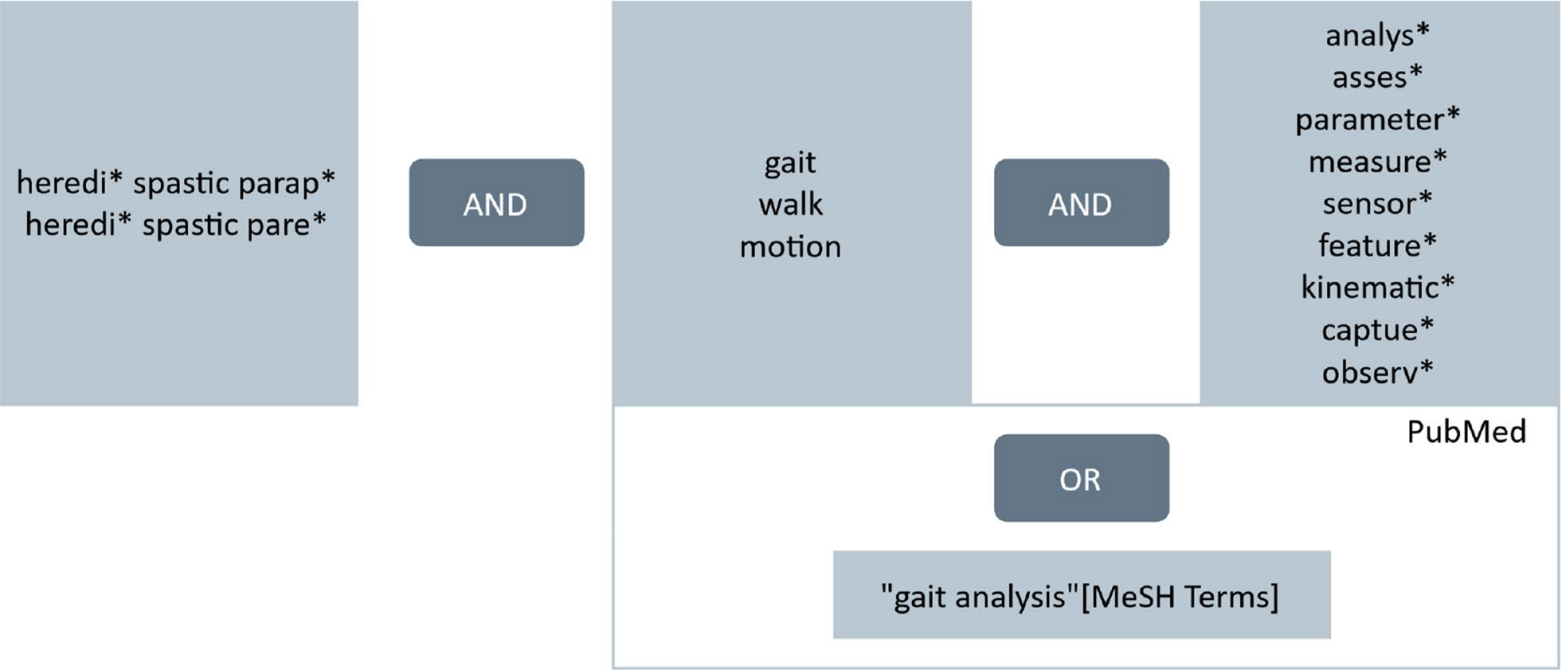
Search strategy. In Web of Science and Scopus the same search strategy was used, for PubMed additionally the mesh term "gait analysis" was included

The search strategy was a combination of HSP terms AND gait terms AND measurement terms (Fig. 1). For PubMed, an additional MeSH term ("gait analysis") was used. The search string for each database is shown in Table S1.

### Selection process

Two independent researchers conducted a literature search based on the inclusion and exclusion criteria. All studies were screened for inclusion by title/abstract and then by full text. In very rare cases of disagreement, a consensus was reached through discussion with an independent third researcher. Eligible studies had to meet the following inclusion criteria: studies with HSP patients of all ages and genders, and an instrumented gait analysis was performed to detect gait-related metrics or improvements of gait patterns. We excluded single case reports and case series, studies that did not primarily focus on HSP (e.g., mixed cohorts with CP), studies that did not focus on gait (e.g. balance, reflex activity, or movement activities other than gait), and studies that did not use instrumented measures (e.g., stopwatch-measured gait test). Importantly, we excluded case and case series reports (study cohort: n < 10) as the cases were interpreted individually instead of performing group analyses, resulting in limited generalizability and the risk of overinterpreting findings from single cases in very heterogeneous disease cohorts.

### Data collection process and data items

Two researchers independently extracted data from the articles using a spreadsheet. Again, discrepancies were solved through discussion between the researchers and, if necessary, a third independent senior researcher was included. Information about author/year, participant characteristics, measurement system, outcome measures, and results. Additionally, the gait metrics were categorized into several groups according to the type: temporal, spatial parameters, kinetic, kinematic, electromyography (EMG), other, and unknown parameters. Parameters related to time and additional distance (spatio-temporal parameters) (e.g., walking speed) were categorized under the spatial parameter category. The type 'unknown' was defined for parameters that were measured but not explicitly mentioned in the study, and 'other' summarizes very specific parameters that could not be classified in any of the defined categories mentioned above. It is important to note that for all parameter types, solely parameters measured during walking were extracted from the articles. For instance, one study assessed EMG during muscle stretching, which was not included in this analysis. Most studies did not provide a parameter definition. For this reason, parameters with a similar meaning were grouped (e.g., trunk tilt, trunk lean, trunk flexion, or trunk and thorax, or lower leg and shank). Also, the nomenclature of the gait parameters in studies differed substantially. Therefore, we used a standardized terminology based on Wolf et al. [21] and Perry [22] (e.g., max hip FlexExt sw refers to the maximum hip flexion–extension angle during the swing phase). Additionally, it was noted whether the gait parameters significantly differentiated HSP patients from controls in observational studies, between treated/untreated groups, before/after treatment in interventional studies, or if no statistical test was performed.

### Study risk of bias assessment

The quality of the included studies was evaluated using the QUADAS-2 tool (Quality Assessment of Diagnostic Accuracy Studies) [21]. This tool consists of four key domains: patient selection, index test, reference standard, and flow and timing. Each domain was assessed in terms of risk of bias and concerns regarding applicability with the signaling questions adapted to align with the aim of this systematic review. In detail, the domains included (a) patient selection: e.g., range of disease severity, exclusion criteria, (b) index test: instrumented gait analysis, (c) reference standard: SPRS or modified Ashworth scale (MAS), and (d) flow and timing: e.g., index test and reference assessed at the same time point. Afterward, two independent researchers performed the quality assessment for all included studies, categorizing each domain as having a 'low', 'high', or 'unclear' risk of bias and applicability concerns [21]. Discrepancies were resolved through discussion to achieve consensus.

## Results

### Study selection

**Fig. 2:**
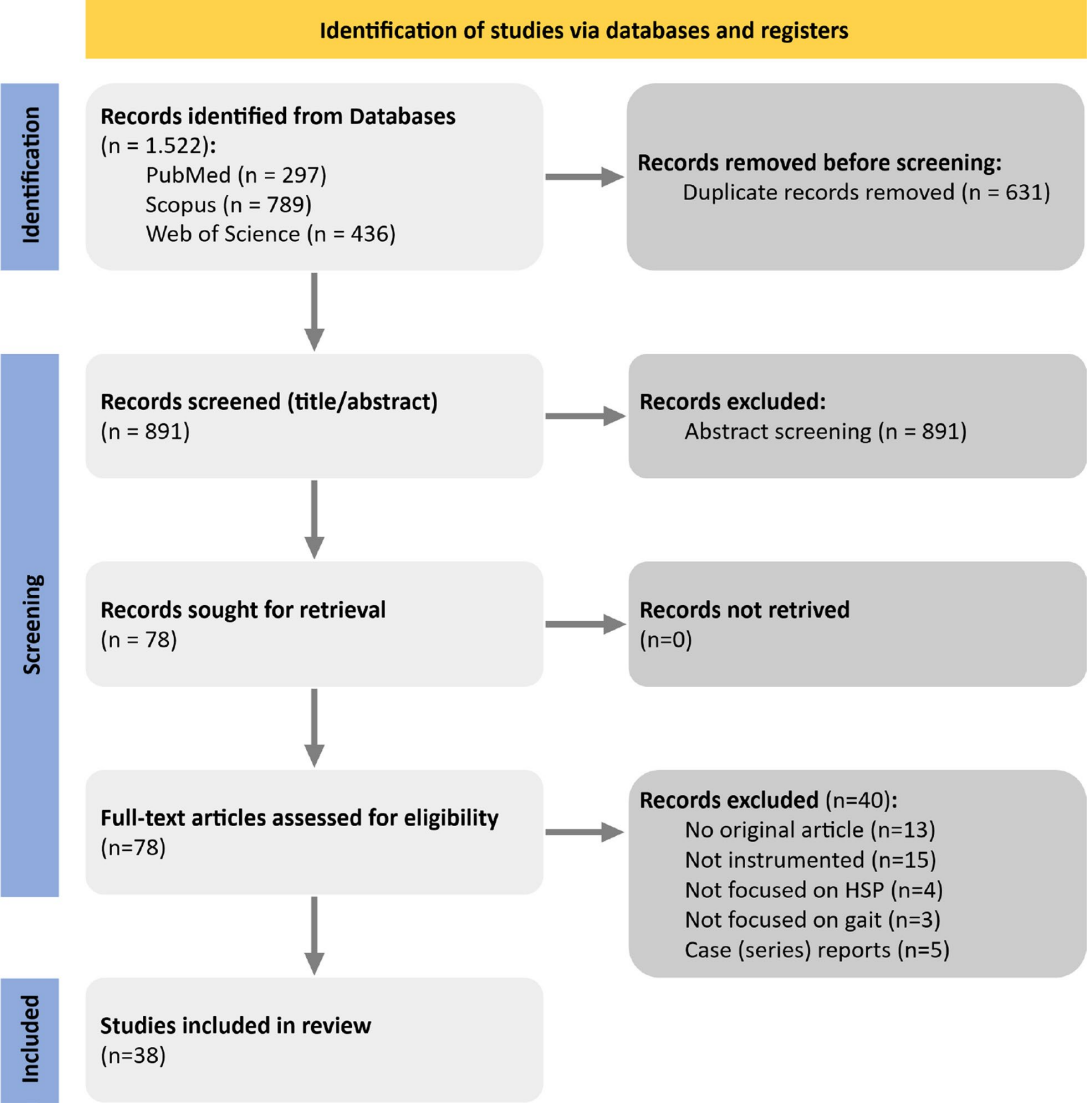
PRISMA flow diagram. This diagram shows the selection process of the included studies. Citation searching was not added to the Figure, as no additional articles were found through this method

Database searching in PubMed, Scopus, and Web of Science resulted in a total of 1.522 articles. After removing duplicates, 891 articles were screened for title/abstract and excluded in case of single case reports and case series, studies that did not primarily focus on HSP, studies that did not focus on gait, and studies that did not use instrumented measures. The remaining 78 articles were checked for eligibility. In total, 38 articles were included in the review, as 40 were removed because they did not meet the inclusion criteria. The PRISMA flow diagram in Fig. 2 shows the detailed selection process.

### Study characteristics

The 38 included articles were published between 2004 and 2024, comprising 29 observational studies and 9 interventional studies. Over half of the studies (n = 25) included a healthy control group, while 7 studies involved other diseases, with CP being the most common. The mean sample size across the studies was 29 HSP patients, ranging from 6 to 112. The mean age of the HSP patients ranged from 9 to 58, except for four studies that did not provide the mean age of participants. Of all studies, 31 included solely adults, 6 included children and young adults, and the remaining paper included a mixed cohort of adults and children [23]. Further details on each study are presented in Table 1. A variety of systems have been used for instrumented gait analysis. The majority of studies used an MOCAP system (26 studies), while one study used an instrumented pressure mat [24], and another used a baropodometric platform [25]. Ten studies used inertial measurement unit (IMU)-based systems, ranging from one to three sensors. Seven of these systems used two sensors at the feet, one used multiple sensors on the lower back and shanks [26], another one on the lower back and feet [27], and one used a single lower back sensor [12]. Out of all the studies included, only three investigated walking on a treadmill [28–30]; all others researched overground walking.

**Table 1:** Study characteristics

Author/year	Study design	Sample size patients (m/f)	Sample size controls (m/f)	Mean age of HSP	Sensor position/MOCAP model	System	Main results	Parameter types
van de Venis et al. 2024 [28]	obs	33 (24/9)	15 (11/4)	489	PIG full body	MOCAP, FP	HSP patients walked with larger gait variability in different parameters compared to controls, reflecting lower gait stability. Clinical measures showed the potential to differentiate fallers from non-fallers in people with HSP	Spatio-temporal parameter, other
Ibrahim et al. 2024 [31]	int	56 (25/31)	–	52	Feet	IMU	Correlations between change of mean and CV temporal parameters with some SPRS subitems. Machine learning models can predict treatment response using baseline clinical and gait measures with a mean accuracy of 66%	Temporal parameter
Beichert et al. 2024 [27]	obs	Lab: 65 (45/20), free: 57(41/16)	Lab: 50 (23/27), free 37(15/22)	Median lab: 53, free: 50	1 lower back + 2 feet	IMU	Spatiotemporal variability measures showed the largest correlations with functional mobility (SPRS mobility), overall disease severity (SPRS, SARA), and activities of daily living (FARS-ADL). The correlations of variability measures with SPRS mobility could be confirmed in mild disease stages and in "simulated free walking"	Spatio-temporal parameter, kinematic, other
Loris et al. 2023 [55]	obs	55 (22/33)	26 (14/12)	47	Feet	IMU	Significant association of gait speed and SPRS progression	Temporal
van de Venis et al. 2023 [32]	int	Training: 18 (14/4), control: 18 (13/5)	–	48	PIG full body	MOCAP	No significant differences in gait measures before and after the training program	Spatio-temporal
Ollenschläger et al. 2023 [9]	obs	50 (24/26)	–	48	Feet	IMU	IMU-based gait analysis system can objectively assess foot elevation in HSP	Spatio-temporal, kinematic, other, unknown
van Gelder et al. 2023 [12]	obs	13 (10/3)	–	54	Lower back + REF (lower back, feet)	IMU + REF (IMU + insoles)	A single lower back sensor is valid for step and event detection of HSP during clinical assessment	Temporal
MacWilliams et al. 2022 [13]	obs	28 HSP	–	n/d (< 18 y)	n/d	MOCAP	Machine learning classifier based on gait measures can classify HSP from bilateral spastic CP	Spatio-temporal, kinematic
Regensburger et al. 2022 [46]	obs	112 (60/52)	122 (49/63)	48	Feet	IMU	Increased stride and stance times, decreased swing duration in HSP compared to HC	Temporal
Joseph et al. 2022 [49]	obs	50 (30/19)	–	48	PIG	MOCAP	HSP gait patterns can be classified based on sagittal knee kinematics, with assistance from sagittal hip and ankle angles	Spatial, kinematic
Lassmann et al. 2022 [14]	obs	p-SPG4: 30 (13/17), p-SPG4 SPRS < 2: 12 (4/8), SPG4: 17 (8/9)	23 (12/11)	p-SPG4: 34, p-SPG4 SPRS < 2:29, SPG4: 48	PIG full body	MOCAP	Objective gait parameters correlate with first symptoms and indicate disease progression from the prodromal to the mild-to-moderate manifest phase	Spatio-temporal, kinematic
van de Venis et al. 2022 [60]	obs	86 (58/28)	–	48	PIG lower body	MOCAP	Spatio-temporal gait parameters vary significantly between groups with different trunk movement levels; increased trunk movements are associated with reduced balance measured by the Berg Balance Scale or Mini-BES test	Spatio-temporal
Coccia et al. 2021 [26]	obs	35 (24/11)	n/d	43	1 lower back + 2 shanks	IMU	Spatio-temporal and kinematics for gait analysis in HSP, show better repeatability and better discriminative power between HC and HSP than sway and APA	Spatio-temporal, kinematic
Martindale et al. 2020 [44]	obs	Pilot: 21 (13/8), validation: 10 (3/7)	–	Pilot: 47, validation: 58	Feet	IMU + REF (Pressure Mat)	Technical validation of a mobile gait analysis system for HSP and correlations between gait parameters and SPRS	Temporal
Martiano et al. 2019 [42]	obs	21 (13/8)	20 (13/7)	46	8 markers lower body parts	MOCAP, EMG	HSP patients show significantly smaller oscillations in distal limb segments (reduced ROM shank, foot, knee, and ankle, and lower foot lift) and distinct some basic temporal patterns in EMG factorization analysis compared to HCs	Spatial, kinematic, EMG
van Vugt et al. 2019 [37]	obs	10 (6/4)	10 (6/4)	54	Cleveland clinical model	MOCAP, FP	The HSP group had slower walking velocity, lower cadence, more time spent in double stance, larger step widths, and greater lateral trunk flexion than HCs	Spatio-temporal, kinematic, kinetic, other
Van Lith et al. 2019 [24]	int	25 (12/13)	–	54	–	Pressure mat	Bilateral botulinum toxin A treatment and subsequent stretching of the hip adductors may improve gait width, gait speed, and reactive lateral stepping in pure HSP	Spatial
Pulido-Valdeolivas et al. 2018 [50]	obs	26 (15/11)	33 (23/10)	9	22 markers lower limbs	MOCAP	Mean pelvic tilt and hip flexion at initial contact are key parameters for differentiating patterns among patient groups	Spatio-temporal, kinematic
Serrao et al. 2018 [51]	obs	26 (12/19)	65 (34/31)	48	22 markers Davis model	MOCAP	Gait patterns formed by the increased step width, reduced ROM ankle, and increased gait variability, can differentiate CA patients from HC and spastic paraplegia or PD patients	Spatio-temporal, kinematic
Martiano et al. 2018 [47]	obs	35 (23/12)	30 (21/9)	49	10 markers lower body	MOCAP, EMG	Widening of muscle activation patterns was observed in HSP using reconstructed spinal maps of MN activity from EMG patterns of 12 muscles	Spatio-temporal, kinematic, EMG
Martindale et al. 2018 [45]	obs	10 (4/6)	–	58	Feet	IMU + REF (PrMa)	Validation of a mobile gait analysis system achieving a mean absolute error of 0.04 s ± 0.03 s for stride time	Spatio-temporal
Van Lith et al. 2018 [34]	int	12 (9/3)	12 (9/3)	51	6 markers (feet)	MOCAP, EMG, FP	HSP patients show overall delayed movement compared to HCs. SAS accelerates tibialis anterior and rectus femoris onsets in both groups, more significantly in HSP. With or without SAS, HSP patients take significantly shorter steps than HCs	Spatio-temporal, EMG
Rinaldi et al. 2017 [40]	obs	23 (17/6)	23 (15/8)	49	Full body Davis model	MOCAP, FP, EMG	Patients differ significantly from HCs in kinematics (step width, ROM knee, and ankle), kinetics (GRF), and EMG data (knee and ankle muscle co-activation indexes)	Spatio-temporal kinematic, kinetic, EMG
Martindale et al. 2017 [43]	obs	21 (8/13)	–	47	Feet	IMU	Segment HSP gait data from a mobile system, into strides using the HMM model achieving a segmentation error of 0.10 s	Temporal
Adiar et al. 2016 [53]	obs	11 (7/4)	28 (14/14)	Median = 11,3	Full body Davis model + 3 trunk	MOCAP	HSP children show increased ROM trunk in the coronal plane and greater trunk and pelvis excursion in the sagittal plane compared to HCs	Spatial, kinematic, other
Serrao et al. 2016 [41]	obs	50 (30/20)	50 (27/23)	48	22 markers Davis model	MOCAP, FP, EMG	HSP patients can be categorized into subgroups of severity depending on the ROM of hip, knee, and ankle joints	Spatio-temporal, kinematic, kinetic, EMG
Riccardo et al. 2016 [25]	int	10 (7/3)	–	40	–	BP	Combined botulinum toxin type A and physiokinesiotherapy in HSP reduces spasticity and improves load distribution (static and dynamic), maintaining benefits for up to 5 months	Spatial
de Niet et al. 2015 [22]	int	15 (12/3)	10 (7/3)	48	PIG full body	MOCAP	Botulinum toxin type-A treatment and subsequent muscle stretching of the calves improved comfortable walking speed and reduced muscle tone in HSP	Spatial
Zhang et al. 2014 [36]	int	9	7	n/d (adults)	Cleveland Clinic marker set	MOCAP, FP	HSP patients have decreased ROM compared to HCs. Hydrotherapy significantly increases walking speed and step length- > it appears that hydrotherapy increases the ability to perform compensatory strategies	Spatio-temporal, kinematic, kinetic
Bonnefoy-Mazure et al. 2013 [38]	obs	10 (5/5)	17 (9/8)	17	Davis protocol	MOCAP	Upper-body kinematics, particularly arm and spine movements, can aid in differentiating HSP from SD, in addition to walking speed, as each condition uses distinct compensation strategies for lower limb alterations	Spatial, kinematic
Marsden et al. 2013 [33]	int	11 (9/2)	11 (9/2)	57	n/d	MOCAP	FES reduces foot drop (increases dorsiflexor torque improves toe clearance and dorsiflexion in the swing phase) and significantly improves walking speed	Spatial, kinematic
Mardsen et al. 2012 [48]	obs	20 (12/8)	18 (11/7)	49	n/d	MOCAP, FP, EMG	Lower knee flexion during the swing phase is linked to reduced knee flexion velocity at the end of the stance phase, with ∼50% of the variance explained by isometric ankle plantarflexion strength and knee extensor passive stiffness	Kinematic, kinetic
Piccinini et al. 2011 [35]	obs	9	15	9	Full body Davis model	MOCAP, EMG	Knee joint parameters can differentiate gait patterns of HSP, HC, and SD children; velocity, step width, and anterior step length differ significantly between the HSP group and HC	Spatio-temporal, kinematic, kinetic, EMG
Wolf et al. 2011 [23]	obs	29 (21/8)	29 (21/8)	23	Lower body gait model	MOCAP	Prolonged hip extension, knee extension, and ankle plantar flexion are indicators for HSP compared to CP	Spatio-temporal, other, unknown
de Niet et al. 2011 [57]	obs	6 (5/1)	13 (6/7)	45	PIG	MOCAP, EMG	Stretch-related mechanisms do not contribute to premature calf muscle activity during stance in UMNS patients. HSP patients show increased calf muscle activity in the first half of stance and lower max Lengthening Velocity	Spatial, EMG, other
Cimolin et al. 2007 [39]	obs	15	20	10	Davis model	MOCAP, FP	HSP children exhibit longer knee hyperextension at midstance compared to CP, with the most significant differences at the knee and ankle joints	Spatio-temporal, kinematic, kinetic
Klebe et al. 2006 [29]	int	22 (11/11)	–	48	14 markers lower body	MOCAP	MPH failed to improve gait and spasticity in SSP/HSP patients over a 6-month period	Spatio-temporal, kinematic
Klebe et al. 2004 (30)	obs	22 (11/11)	18 (10/8)	48	14 markers lower body	MOCAP	HSP and SSP patients show significantly lower walking speed, stride length, step height, and ROM knee compared to controls; gait patterns are similar between SSP and HSP cases	Spatio-temporal, kinematic

*obs* observational study, *int* interventional study, *PIG* Plug-In-Gait marker model, *REF* reference system, *p-SPG4* prodromal SPG4, *APA* Anticipatory postural adjustment, EMG Electromyography *n/d* not defined, *IMU* inertial measurement unit, *MOCAP* Motion capture system, *FP* force plates, *CV* Coefficient of variation, *SARA* Scale for the Assessment and Rating of Ataxia, *FARS-ADL* activities of daily living subscore of the Friedreich Ataxia Rating Scale, *BP* baropodometric platform, *PrMa* Pressure Mat, *SPRS* Spastic Paraplegia Rating Scale, *HSP* hereditary spastic paraplegia., *ROM* Range-of-motion, *EMG* electromyography, *GRF* ground reaction force, *HMM* hidden Markov model, *SSP* sporadic spastic paraplegias, *MPH* Methylphenidate, *SAS* startling acoustic stimulus, *y* years, *SD* spastic diplegia, *FES* Functional Electrical Stimulation, *CP* cerebral palsy, *UMNS* upper motor neuron syndrome, *HC* healthy subjects, *APA* Anticipatory postural adjustment, *CA* cerebellar ataxia, *PD* Parkinson’s disease, *MN* Motor neurons

The interventional studies investigated a variety of interventions, including botulinum toxin type A [31], a combination of botulinum toxin and daily stretching exercises [22, 24], a combination of botulinum toxin A and physiokinesiotherapy [25], treatment with methylphenidate [29], gait-adaptability on C-Mill [32], hydrotherapy, Functional Electrical Stimulation (FES) [33], and a startling acoustic stimulus simultaneously paired with an imperative stimulus [34].

### Risk of bias in studies

**Fig. 3:**
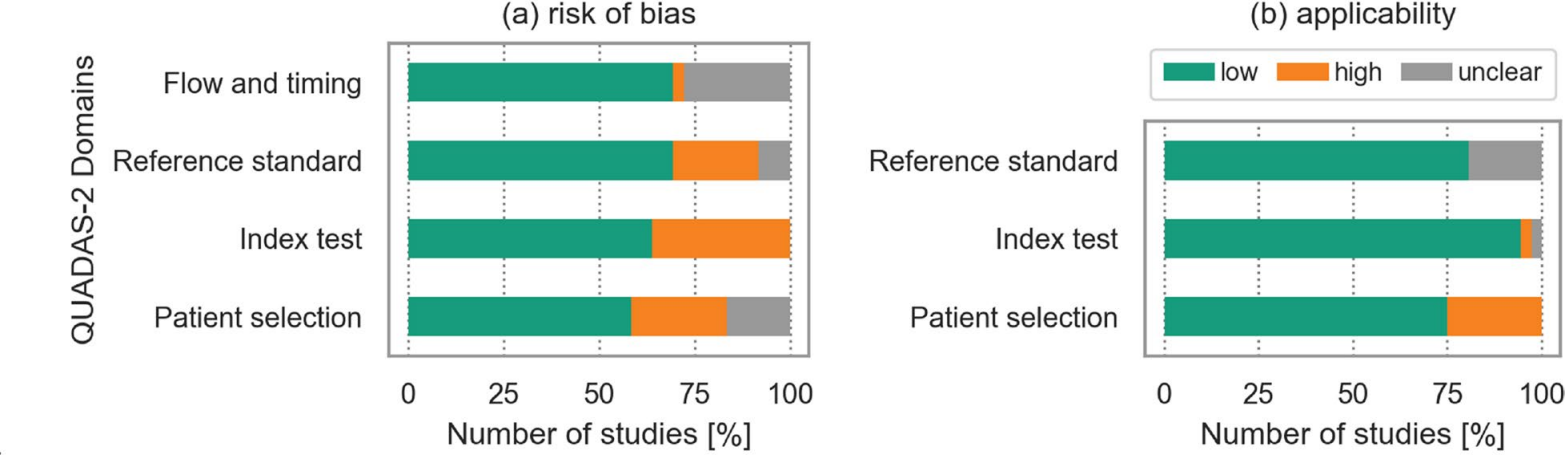
Summary of the quality of all included studies using the QUADAS-2 tool (**a**) risk of bias (**b**) concerns regarding applicability. *QUADAS*-2 Quality Assessment of Diagnostic Accuracy Studies

A summary of the quality of the included studies, rated by the QUADAS-2 scale, is presented in Fig. 3; details by study are given in Table S2. In total, 7 out of the 38 included studies were rated ‘low’ in all 7 domains concerning both risk of bias and concerns regarding applicability. In contrast, one study was rated with ‘high’ or ‘unclear’ in all domains. For another 15 studies, 6 domains were rated ‘low’ and the remaining domain ‘unclear’ or ‘high’. Further, 7 studies were identified in which 5 domains were rated ‘low’ and the remaining 8 were assessed with ‘low’ quality in 2 or 3 domains.

Eleven studies were classified as having a ‘low’ risk of bias in all assessed risk of bias domains. Patient selection had the highest risk of bias with 58% of the studies showing a ‘low’ risk of bias, 25% ‘high’ and the remaining 17% ‘unclear’. The index test of the risk of bias assessment was the domain that had either a ‘low’ or ‘high’ risk of bias.

Twenty-three studies were assessed as having ‘low’ concerns regarding applicability across all corresponding domains. ‘High’ concerns regarding applicability occurred in the domain of patient selection (25%); for the other two categories, most of the studies had ‘low’ concerns or were rated as ‘unclear’.

### Digital outcome measures

**Fig. 4:**
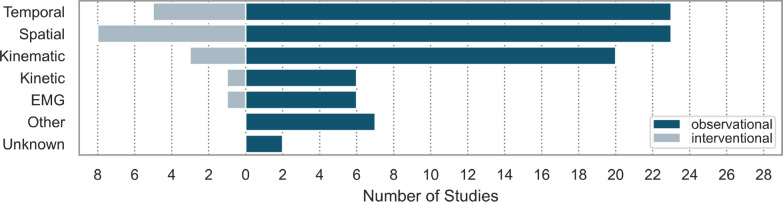
Gait parameter types used in the different interventional and observational studies; ‘other’: parameters which could not be classified in one of the categories; ‘unknown’: more parameters were measured, but they were not explicitly mentioned. *EMG* Electromyography

The digital outcome measures observed in HSP patients were categorized into several groups: spatial-, temporal-parameter, kinetic, kinematic, EMG, other [e.g., total energy consumption (TEC)], and unknown parameters. Figure 4 provides an overview of the number of studies utilizing parameters of each type. These parameters were analyzed separately for both observational and interventional studies. Parameters classified as ‘other’ could not be assigned to any of the predefined groups. The ‘unknown’ category occurred in two studies [9, 35]. Among observational studies, spatial and temporal parameters were the most commonly used, with 23 out of 29 studies incorporating both. In interventional studies, spatial parameters were the most frequently reported, as they were included in every study except one.

#### Observational studies

**Fig. 5:**
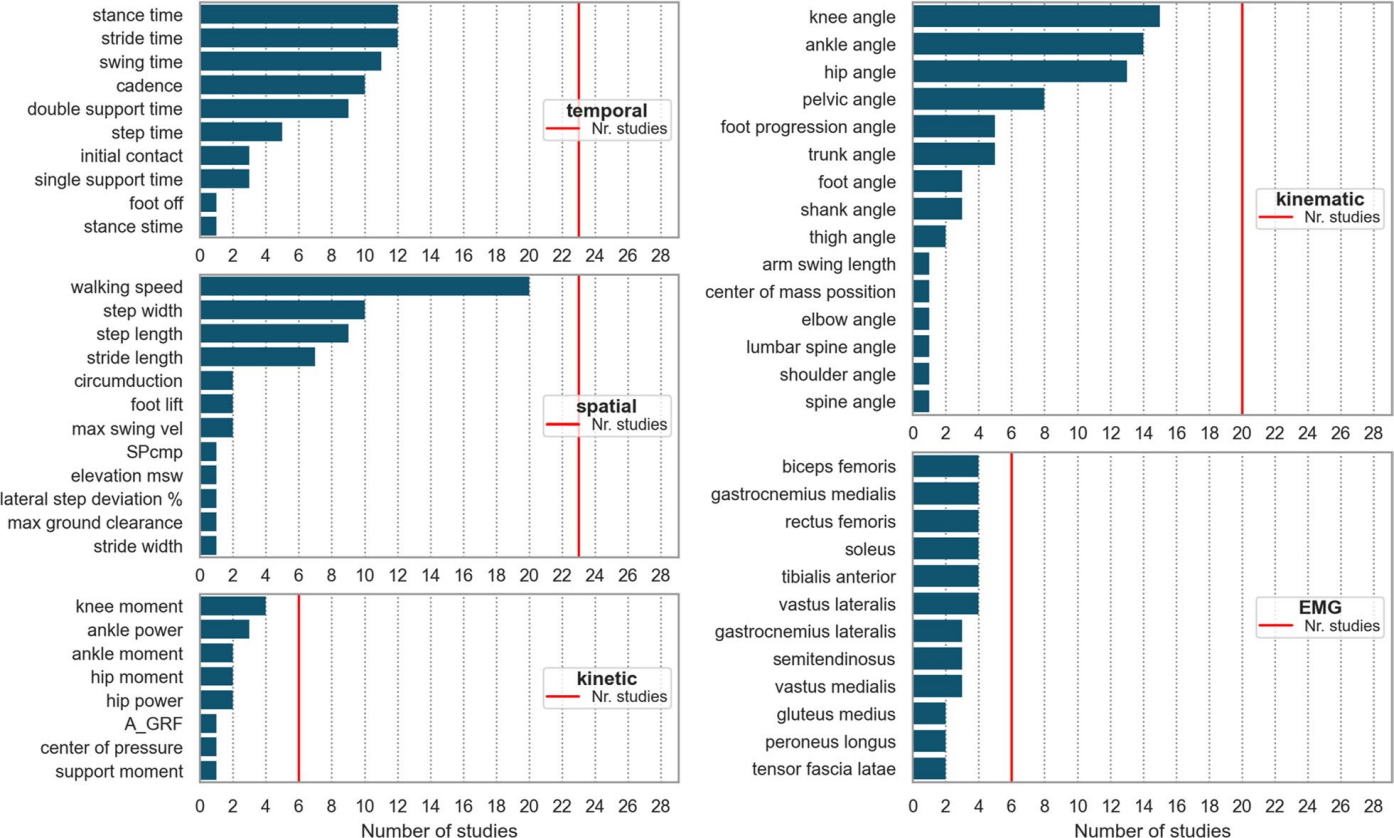
Single gait parameters of all parameter types included in the observational studies. As parameters categorized in the ‘others’ group were used sporadically in a few studies, they were not displayed in this figure. Red lines indicate the number of studies that included the specified parameter type in total. For spatio-temporal parameters, no derived parameters are displayed. Kinematic, kinetic, and EMG parameters were grouped based on the involved joint/segment/muscle. *EMG* Electromyography, *SPcmp* spatial variability composite measure, *max* maximum, *A* area under the curve, *GRF* ground reaction force, *msw* mid swing phase

A total of 29 observational studies were conducted, examining the parameters presented in Fig. 5. The two most frequently reported temporal parameters were stride time (reported in 11 studies) and cadence (reported in 10 studies), followed by swing and stance duration (each reported in 8 and 9 studies, respectively). Notably, 44% of the temporal parameters were solely reported once. Among the spatial parameters, walking speed was the most commonly used, observed in 20 out of 23 studies that reported spatial parameters, followed by step width, reported in 10 studies. The majority of spatial parameters were only observed once. Regarding kinematic parameters, 17 different angles, velocities, and positions were measured, with generic parameters being calculated for each. The most frequently observed ones were knee, ankle, and hip angles, included in 15, 14, and 13 studies, respectively. For kinetic parameters, 10 different parameters were measured. The most commonly used kinetic parameters were ankle power and knee moment, each reported in three studies. In terms of EMG, 12 different muscles were investigated. Most frequently, the following 6 muscles were measured: rectus femoris, vastus lateralis, tibialis anterior, gastrocnemius medialis, biceps femoris, and soleus.

#### Interventional studies

**Fig. 6:**
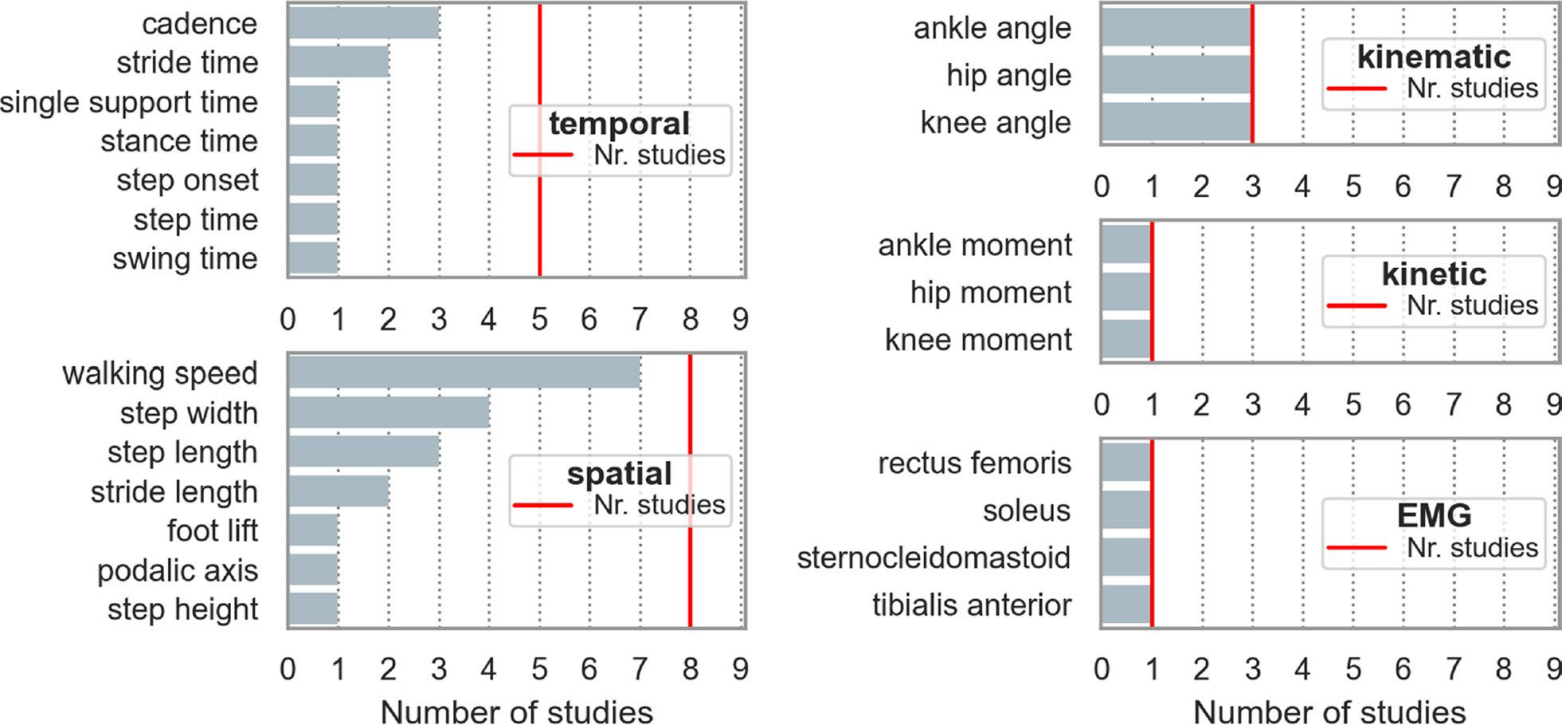
Single gait parameters of all parameter types included in the interventional studies. Red lines indicate the number of studies that included the specified parameter type in total. For spatio-temporal parameters, no derived parameters are displayed. Kinematic, kinetic, and EMG parameters were grouped based on the involved joint/segment/muscle. *Nr*. number, *CV* coefficient of variation

The 9 interventional studies included various parameter types: 10 different temporal parameters, 8 spatial parameters, 3 different joint angles (kinematic parameters), moments (kinetic parameters), and 4 different muscles (EMG). These individual parameters are presented in Fig. 6. The most frequently observed temporal parameter was cadence, reported in 3 [29, 32, 36] out of 5 studies that included temporal parameters. All other temporal parameters, except stride time (n = 2) [31, 32], were only reported in one study. Walking speed, measured in 7 out of 8 studies that included spatial parameters, was the most frequently reported spatial parameter. Regarding the kinematic parameters, 3 studies reported generic parameters for the hip, knee, and ankle angles. Concerning kinetic parameters, one study reported parameters of the hip, knee, and ankle [36]. One study measured EMG [34], but the authors did not include muscle activity. Instead, they focused on calculating muscle offsets during gait initiation.

### HSP-relevant digital outcome measures

In the following, solely parameters were reported that showed significant differences in at least one study (i) between HSP patients and healthy controls (observational studies), (ii) between treated and untreated groups (interventional studies), or (iii) comparing before and after treatment (interventional studies).

#### Observational studies

**Fig. 7:**
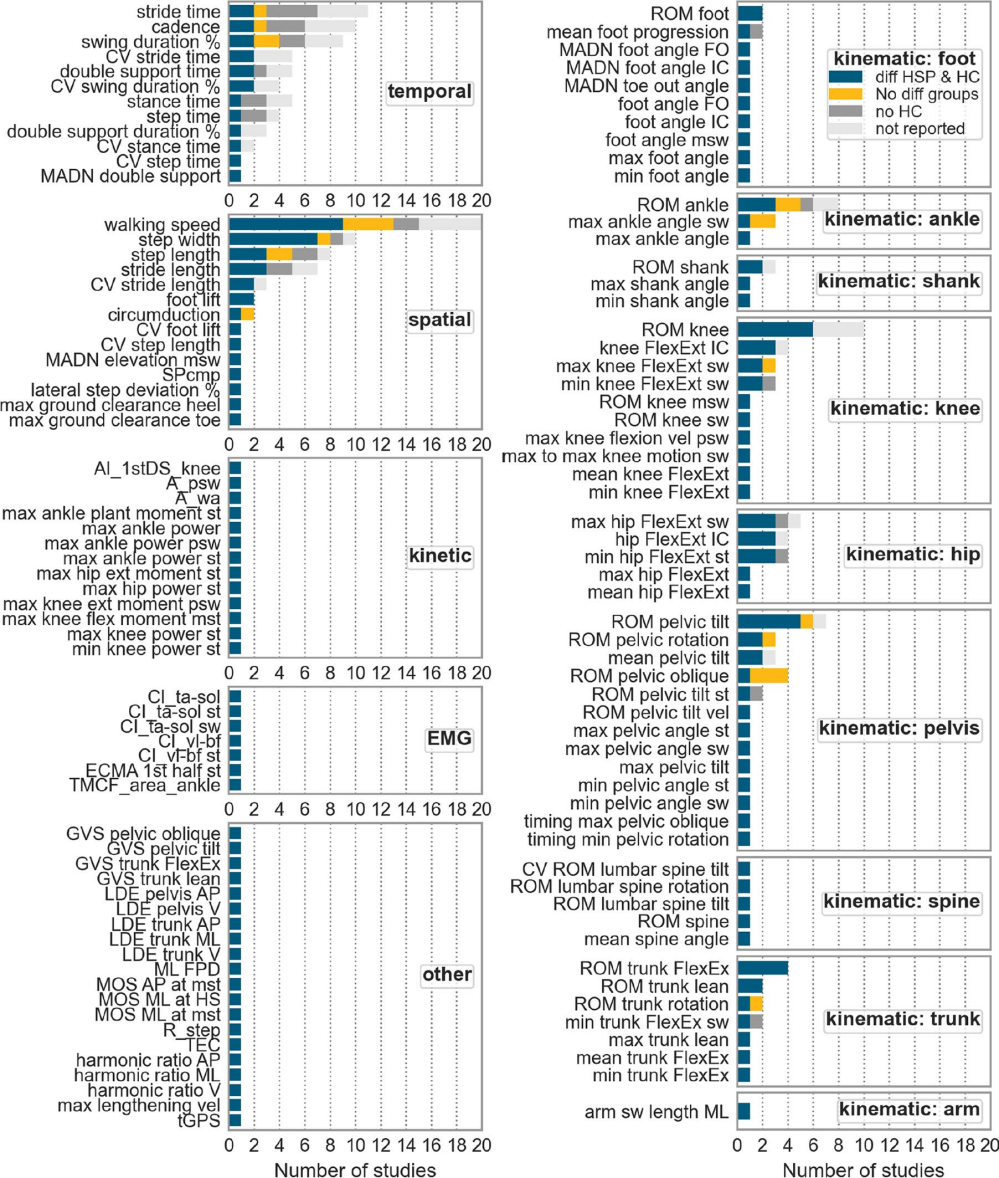
Gait parameters of all gait parameter types, differentiated at least once between patients and healthy controls, are displayed in the figure. One study used median for outcome measures instead of mean [27]. *diff* difference, *HSP* hereditary spastic paraplegia, *HC* healthy controls, *CV* coefficient of variation, *max* maximum, *ext* extensor, *A* area under the curve, *wa* weight acceptance phase curve, *st* stance phase, *mst* mid stance phase, *psw* preswing phase, *AI* angular impulse, *DS* double support, *CI* coactivation index, *ta* tibialis-anterior, *sol* soleus, *TMCf* time-varying multi-muscle co-activation function, *sw* swing phase, *vl* vastus lateralis, *bf* biceps femoris, *ECMA* Early Calf Muscle Activity, *R* fraction of mechanical energy, *MOS* Margin of Stability, *ML* medio-lateral, *GVS* Gait Variable Scores, *vel* velocity, *TEC* total energy consumption, *tGPS* Gait profile Score including trunk kinematics, *HS* heel strike, *FlexEx* flexion/extension, *AP* Anteroposterior, *ROM* range-of-motion, *IC* initial contact, *min* minimum, *msw* midswing phase, EMG Electromyography

A total of 365 different gait parameters were reported in observational HSP studies, but only 214 were used to compare HSP patients and healthy controls (see Table S3). Results show that 123 of 214 parameters significantly differentiated between both groups (Fig. 7). Walking speed was identified as the most frequently differentiating parameter in 9 studies [14, 23, 27, 30, 35, 37–39], followed by step width in 7 [28, 30, 35, 37, 39–41] and knee range-of-motion (ROM) in 6 [23, 30, 38, 41–43]. Spatio-temporal parameters often presented significant results across multiple studies. Many kinematic measures were used, with over 26% providing significant results in more than one study. In contrast, parameters related to kinetics, EMG, and others differed significantly solely in one study.

#### Interventional studies

**Fig. 8:**
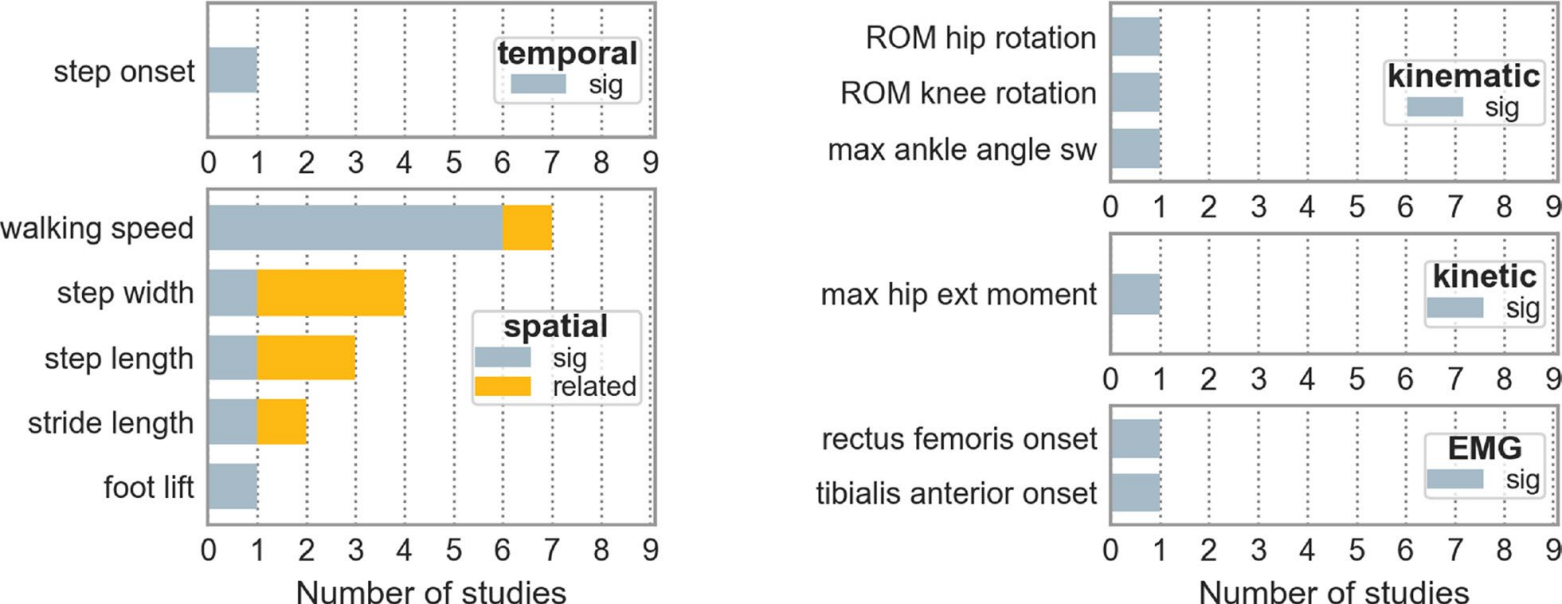
Parameters related to therapy response of HSP patients are displayed in the figure. The number on the x-axis shows in how many studies these parameters were observed to be related to therapy response. *ROM* range-of-motion, *max* maximum, *sw* swing phase, *EMG* Electromyograph

Among the 44 gait parameters measured in interventional studies, 12 demonstrated a treatment effect through various interventions (overview in Table S4). Walking speed was the most frequently reported parameter, showing improvements in 6 [22, 24, 25, 33, 36] out of 9 interventional studies. All other parameters showed improvements in only one study. A detailed overview is presented in Fig. 8.

## Discussion

This systematic review aimed to summarize the outcome measures of instrumented gait analysis, including inertial sensors and MOCAP systems, and to identify the most important HSP-specific digital outcomes. To date, instrumented gait analysis has been used in observational and interventional studies in adults and children, providing different metrics to characterize gait patterns in HSP patients. In the following, we describe the most robust parameters and identify urgent needs for future studies.

### Relevance of stationary and mobile systems for gait analysis

Most studies (n = 26) were conducted in a controlled laboratory environment, using different stationary MOCAP systems. Significantly fewer studies (n = 10) investigated different mobile sensor systems. MOCAP systems offer a wide range of parameters based on recordings in a stationary clinical setting. As a result, MOCAP systems are essential for precisely characterizing HSP gait patterns. However, they are limited to the hospital setting, whereas wearable sensor systems have the potential to be used in the home environment of HSP patients, reflecting real-world gait patterns. Interestingly, one study simulated real-life walking in a hospital setting [27]; however, this approach provides limited relevance for gait patterns recorded in the patients’ home environment. Consequently, mobile, sensor-based systems are urgently needed, but they must provide technically robust and clinically meaningful measures. Hence, sensor-based systems need to undergo stringent technical and clinical validation. A proposed single-sensor approach seems to offer a reliable method for identifying steps in HSP [12]. Another IMU-based system with sensors fixed on the instep of both feet was technically and clinically validated [43–45]. The temporal parameters appear to validly reflect the gait impairment of HSP patients in the hospital [46]. Significant correlations between temporal parameters, including coefficients of variation (CV)s and the SPRS, were detected, as well as with SPRS gait subitems, and all parameters were also significantly correlated with the Falls Efficacy Scale (FES-I) (patient-reported fear of falling) [46]. A three IMU-sensor based approach also showed recently in a cohort of SPG7 patients additional correlations between kinematic parameters of the lumbar spine and feet with the SPRS [27], however, the sensor system was not adapted for HSP.

Most IMU approaches, except for 3 studies [9, 26, 27], have solely considered temporal parameters and were not validated for other parameters in HSP. Two studies analyzed more than one kinematic parameter using an approach based on three IMU sensors [26, 27]. One of the studies has also shown that the spatio-temporal and kinematic parameters, previously observed to differentiate between HSP and healthy controls, demonstrate excellent test–retest reliability [26]. The other study analyzed kinematic parameters based on sensors placed on the lumbar spine and feet, showing that an additional sensor may provide further information [27]. These results indicate that additional IMU-based parameters require technical and clinical validation in future studies.

Previous studies have shown significant correlations between spatio-temporal and kinematic parameters and the SPRS [14, 27, 42, 44, 46, 47]; the single parameters are presented in Table S5. These studies demonstrate the clinical relevance of instrumented gait analysis and the added value for HSP, indicating that capturing those parameters in hospital settings and especially monitoring them in everyday life is meaningful. This is of particular importance as there is a lack of studies investigating daily life mobility in HSP. In addition, except for the study mentioned above, there is a lack of evidence for the potential need to use multiple sensors to better reflect full-body movements instead of monitoring impaired movement patterns from a single body position. This aspect should be investigated in future studies.

### HSP relevant digital outcome measures

Numerous studies have used instrumented gait analysis in HSP (76% observational, 24% interventional) to characterize gait patterns [29, 35, 39–41, 48], subgroup HSP patients [23, 41, 47, 49, 50], distinguish HSP patients from other diseases with similar characteristics [13, 23, 35, 38, 39, 51], and evaluate the effect of treatment [22, 24, 25, 30–34, 36].

#### Spatio-temporal and kinematic parameters

Based on the included studies, the spastic gait pattern of HSP patients significantly differs from those of healthy controls in spatio-temporal and kinematic parameters. HSP patients, both children and adults, show reduced walking speed, stride length, cadence, and ROM of the distal joints and segments (such as sagittal ankle and knee angles) compared to healthy controls [29, 35, 41, 42, 48]. From a clinical perspective, it is reasonable that these parameters distinguish between HSP and healthy controls since, e.g., reduced walking speed is a consequence of impaired walking, and a decreased ROM knee reflects leg spasticity in HSP patients.

Walking speed was identified in 9 [14, 23, 27, 30, 35, 37–39] out of 20 studies as an important parameter to differentiate between HSP patients and controls. Four studies found no difference but used speed-matching to avoid gait speed as a confounding factor [40–42, 48]. Since walking speed affects gait patterns, including spatio-temporal parameters, joint kinematics, and joint kinetics [52], it should be accounted for when comparing healthy controls and HSP patients. This consideration was made in one interventional and six observational studies [40–42, 47, 48, 51] and should be taken into account while interpreting the results. However, this can be challenging due to the variability in walking speed within the patient groups.

The ROM knee was the most frequently observed parameter distinguishing HSP patients from healthy controls, with Martino et al. [47] highlighting its importance for characterizing HSP gait. Marsden et al. [48] observed that adults with HSP walk stiff-legged with less knee flexion during the swing phase. Additionally, ten different knee parameters significantly distinguished patients from controls. However, ROM ankle (n = 8), ROM hip (n = 8), and ROM pelvic tilt (n = 7) were analyzed almost as frequently as ROM knee. However, the ROM ankle seems less relevant as the results were contradicting. In contrast, ROM trunk Flexion/Extension was significantly increased in 4 studies [37, 38, 41, 53]. Increased trunk movements were also observed in frontal and transversal planes [41, 53], indicating that HSP patients compensate for altered lower body movements with trunk and pelvic movements [38]. Interestingly, ROM hip did not distinguish between groups, although studies noted differences in specific hip angle parameters (see Fig. 7).

These findings indicate that novel parameters are needed for characterizing the gait deviation of the hip, such as the max hip Flexion/Extension swing phase and min hip Flexion/Extension stance phase, as both differentiated three times. In 5 out of 6 studies, the ROM pelvic tilt distinguished between HSP patients and healthy controls. Notably, the sole study that showed no differences involved adults [41], while the others involved children [35, 38, 39, 53] or a mixed cohort [23]. Figure S1 shows kinematic variables separately for children and adults, indicating that a larger variety of kinematic parameters were measured for children. Conversely, more spatio-temporal parameters were observed for adults (Figure S2). It is important to note that children walk at a slower pace with shorter stride lengths than adults [54], which may also influence kinematics. Moreover, the progression and clinical characteristics of childhood-onset HSP differ from those of adult-onset HSP. This indicates the need for more detailed studies of spatio-temporal and kinematic parameters in both adults and children.

Interestingly, reduced ROM foot segments, as well as decreased foot lift and max ground clearance [14, 42], appear to be clinically relevant parameters. Lassmann et al. [14] reported reduced ROM foot segment and max ground clearance even in prodromal HSP patients. In total, numerous kinematic parameters were measured; however, out of 251 kinematic parameters, only 39 (14%) were calculated more than twice (Table S3), confirming a lack of clearly defined outcome measures. These findings indicate that ROM knee and foot are crucial digital measures for HSP. However, to establish potential endpoints for clinical studies, it is necessary to conduct multicenter studies, acknowledging the limited sample sizes of this rare disease. Notably, three more recent studies used a multicenter design to reach higher statistical power [27, 31, 46].

In a longitudinal study, Loris et al. [55] observed that some temporal parameters appear to reflect disease progression. Stride time, stance time, and relative swing duration demonstrated significant correlations with the sum of the functional subitems of the SPRS [55]. Apart from this study, only one case series focused on longitudinal instrumented gait data. They observed that early-onset gait deviations tended to improve until adolescence and decline in adulthood [56]. Two additional studies analyzed longitudinal data in a sub-cohort of their patients [44, 46]. A more recent study observed that sensor-based gait parameters reflect disease progression in a cohort of 11 HSP patients [46]. In general, there is a huge lack of longitudinal studies using instrumented gait analysis in HSP. Future investigations should focus on detecting disease progression and therapy response in repeated measure designs as well as stringent application of patient and clinician reported anchor parameters.

#### EMG and kinetic parameters

EMG and kinetics were not frequently studied in HSP. Only 6 out of 29 observational studies examined EMG patterns [35, 40–42, 47, 57] and kinetics [33, 35, 37, 39–41]. One EMG study included children but described the raw data only, reporting decreased rectus femoris activation in children with HSP [35]. EMG studies in adults found increased and premature calf muscle activity during the first half of stance [57]. In addition, significantly increased co-activation of the antagonistic ankle [35, 40–42, 47, 57] and knee muscles [40] correlated with Ashworth scores, indicating a link to lower limb spasticity [40]. Complex methods were used in two studies to analyze spinal locomotor output [47] and assess locomotor coordination [42] in HSP to better understand its abnormal locomotor pattern.

Concerning kinetics, solely moment and power of the ankle, knee, hip, and area under the ground reaction force curve (A_grf) were considered, whereas these parameters were observed only once. The findings show a tendency of lower values for A_grf [40] and lower ankle power in different subphases [35, 39, 48]. Kinetics are essential for a complete understanding of gait dysfunctions [58]. When combined with kinetics and kinematics, EMG may be a valuable tool for identifying the most efficient treatment method [15]. Further studies are needed to understand the impact of EMG and kinetics in adults and children with HSP.

#### Relevance of subgrouping/clustering in HSP

Gait parameters were not solely used to compare HSP patients with healthy controls, but interestingly also for subgrouping and gait pattern classification due to the heterogeneity of the disease [1]. Wolf et al. [23] conducted a cluster analysis on the sagittal gait kinematics of HSP and CP patients, resulting in five clusters: 'crouch gait', 'recurvatum gait', 'stiff knee gait', 'jump knee gait', and 'norm-like gait'. These clusters were similar to the CP gait classification system by Sutherland and Davids [23, 59].

Pulido-Valdeolivas et al. [50] conducted a clustering analysis and identified six walking patterns in children with HSP. Five of these patterns were similar to the findings of Wolf et al. [23]. Mean pelvic tilt and hip flexion at initial contact were the most important parameters for differentiating patterns. In the study of Joseph et al. [49], two physiotherapists were able to classify adult HSP patients into the groups proposed by Wolf et al. [23] and showed significant differences in ankle, knee, and hip angles using Statistical Parametric Mapping (SPM) between groups [49]. Serrao et al. [41] identified three distinct HSP walking patterns based on ROM differences: subgroup one with reduced ROM hip, knee, and ankle; subgroup two with reduced ROM knee and ankle; and subgroup three with increased ROM hip. These subgroups also differed in spatio-temporal parameters and correlated with disease severity (SPRS) [41]. Martino et al. [47] used the identical subgrouping method and observed subgroup differences in the spinal maps of motor neuron (MN) activation.

Ollenschläger et al. [9] used a machine learning classifier to detect reduced foot elevation in adults with HSP, achieving accuracy close to clinical assessments (88%). Van de Venis et al. [60] grouped patients by increased trunk movements and found an association of these with reduced balance capacity. However, in a more recent study, the same authors did not observe differences in gait parameters between fallers and non-fallers, whereas distinctions in clinical and functional scales existed [28].

Due to the phenotypic heterogeneity of the disease, subgrouping of HSP patients may be a useful method to comprehensively characterize HSP-specific gait patterns. This method could also help to define an individualized treatment for the affected patients and the best treatment option for each patient group. Previous research indicated that a classification system resembling an adapted CP classification may be appropriate for HSP as well.

#### Instrumented gait patterns differentiate disease entities

Patients with childhood-onset HSP may resemble those with CP, leading to a misdiagnosis during childhood [1, 13, 35, 39]. However, HSP is progressive, and treatment approaches may differ [23, 38]. Hence, instrumented gait analysis might be a useful tool to distinguish between these diseases, especially when genetic analyses remain negative. Several studies have reported gait parameters distinguishing HSP from CP [13, 23, 35, 38, 39], cerebellar ataxia (CA), and Parkinson’s disease (PD) [51].

Cimolin et al. [39] aimed to assess differences between children with HSP and CP. Interestingly, they observed differences in the knee and ankle joints but not in spatio-temporal parameters. Both groups showed knee hyperextension in midstance, but solely the HSP group had a prolonged stance duration [39]. Piccinini et al. [35] also observed significant differences in knee kinematics and additional knee kinetics, supporting the importance of the knee in differentiating these two pathologies. Wolf et al. [23] performed a cluster analysis and identified some gait characteristics indicative of HSP: prolonged hip extension, knee hyperextension, ankle plantar flexion, and large trunk tilt velocities. However, they could not clearly distinguish HSP from CP patients due to the heterogeneity of these diseases. Additionally, they found that more HSP patients showed a recurvatum knee (e.g., prominent knee extension or hyperextension in mid-stance) compared to CP [23].

Importantly, in contrast to the previous studies, Bonnefoy-Mazure et al. [38] included upper body motions in their research and found that HSP patients compensated with a rapid spine tilt, while CP patients used their arms, resulting in increased shoulder elevation and elbow flexion compared to HSP. MacWilliams et al. [13] used a machine learning classifier to differentiate between patient groups (HSP and CP) based on gait and physical exam variables, achieving high specificity and sensitivity. In contrast to the other studies, Serrao et al. [51] compared gait patterns in patients with degenerative neurological diseases (HSP, CA, and PD) and found that the ROM ankle was included in all clusters when clustering sets of parameters for differentiating diseases.

In summary, knee kinematics and upper body motions play a major role in differentiating these diseases; however, due to small sample sizes, more research, especially in upper body motions, is needed. These studies indicate that instrumented gait analysis is an important objective measure to detect intra- and inter-disease differences.

#### The role of instrumented gait measures in interventional studies

Instrumented gait analysis was also used to evaluate the effect of treatment in HSP. However, 24% (n = 9) of the included studies only had an interventional study design, and none involved children. While 365 different parameters were used in observational studies, only 44 were measured in interventional (8%) studies, and only one included EMG [34] and kinetics [36]. More interventional studies were performed; however, they did not use instrumented gait measures [16], potentially because no clinically relevant digital outcome parameters are defined yet. Ibrahim et al. [31] used IMU-based gait metrics in a multicenter interventional study representing a promising example for future trials. This multicenter study involving the largest interventional cohort of 56 HSP patients found significant correlations between gait parameters and clinical scores regarding treatment effects and, for the first time, predicted treatment response using machine learning models.

In the 9 interventional studies, 27% of the measured parameters showed improvement. Given that progressively worsening gait deviations are one of the main symptoms of HSP [2, 3], gait analysis has the potential to be a valuable objective indicator of improvement in interventional studies. Future research should, therefore, aim to further identify clinically relevant outcome measures. However, since those studies were conducted with small sample sizes (average of 21), the strength of expression of these gait parameters is limited and should be interpreted carefully. Therefore, further studies are needed to define relevant digital outcome measures for HSP as potential endpoints for clinical studies.

In addition, simulations of HSP gait may be used to predict interventional outcomes, as a recent study did a computer simulation with a neuro-muscular model of human walking [61]. These are promising steps toward clinically relevant digital gait outcomes in HSP.

#### Quality of the included studies

The quality of the studies was assessed using the validated QUADAS-2 tool. The highest risk of bias was obtained in the domain of patient selection, mainly because of an unknown (n = 10) or slight (n = 7) disease severity. Slight was defined as SPRS < 16 [62] or MAS < 1.5 [30] as defined. The MAS was used if no SPRS was applied, as the MAS also appears to be suitable for classifying the severity of spasticity in HSP [30].

The ‘high’ risk of bias in the reference standard domain was primarily due to instances where the SPRS or MAS was either not assessed or not reported. In the flow and timing domain, an ‘unclear’ risk of bias was either due to the missing reference standard test or insufficient information regarding the order or execution of the gait test. The ‘high’ risk in the index test domain was associated with a lack of parameter explanation or the conduction and analysis of the gait assessment (e.g., only strides of one side were used or walking on a treadmill).

In conclusion, most of the included studies were rated as having a 'low' risk of bias and low concerns regarding applicability. Therefore, the general quality of studies in the investigated research field is good. However, due to distinct studies revealing high concerns, the interpretability of the results is limited.

## Limitations

This review has some limitations that should be considered when interpreting the findings. Some of our exclusion criteria may have excluded potentially relevant studies, as we did not consider case and case series reports. For feasibility reasons, we limited the included records to articles solely written in English. Further, the sample size of the included studies was rather small, as 79% of the studies consisted of less than 50 participants, but this was expected for a rare disease such as HSP. Additionally, different instrumented systems, disease stages, and genotypes were included. Some papers consisted solely of HSP patients with a pure form, others with mixed cohorts, contributing to the heterogeneity of the results described above. Notably, some papers excluded patients who were not able to walk without walking aids, so more advanced disease stages were excluded in more than 50% of the studies. In addition, several studies were conducted by identical working groups, so the patient cohorts in different studies could include identical patients. Since patient cohorts (high risk of bias for patient selection), test settings (e.g., pre-defined gait speed vs. preferred gait speed), and equipment for instrumented gait analysis were highly heterogeneous, it was decided to report the results without performing a meta-analysis to avoid inadequate interpretations. The risk of bias assessment in this review was focused on the diagnostic tool (instrumented gait analysis) rather than the study designs of the included studies, and the systematic review was not prospectively registered. We decided not to use the Grading of Recommendations, Assessments, Development and Evaluation (GRADE) system to detect the quality of evidence in existing literature, as this method appeared not appropriate for our aim. Future systematic reviews with meta-analysis should consider using GRADE.

## Conclusion and recommendations for further research

In conclusion, this systematic review highlights the important role of digital outcome measures of instrumented gait analysis in understanding and characterizing gait patterns in HSP. The findings indicate that while stationary MOCAP systems provide insights into the gait characteristics of HSP in controlled lab environments, there is a need for validated inertial sensor systems to capture daily life walking patterns. It appears that gait analysis is a promising tool for detecting intra- and inter-disease differences in HSP. Spatio-temporal and kinematic parameters are widely used and have shown significant correlations with disease severity. Notably, three key parameter groups are recommended: (i) evidence-based: walking speed and ROM knee, since they have been identified to distinguish HSP patients from healthy controls, but it should be considered that both parameters are rather general than disease-specific and that a change in gait speed impacts other gait parameters. (ii) Promising, further research needed: foot lift, ROM foot, and gait variability, since those parameters reflect disease severity in terms of reduced foot movements due to increased spasticity and increased balance deficits represented by larger gait variability, and (iii) contradicting results, but with potential clinical relevance: ROM hip. Furthermore, this review shows the importance of validating additional parameters and developing clinically relevant digital outcome measures to improve the assessment of disease progression and therapy response in HSP.

Despite the potential of instrumented gait analysis, there is a lack of studies, particularly regarding the longitudinal assessment of gait changes and the integration of EMG, kinetic data, and upper body parameters. In addition, it is important to gain a deeper understanding of gait patterns for different disease onsets and the differentiation between children and adults with HSP. Future research is needed for three aspects: (i) structured longitudinal multicenter studies with larger sample sizes to establish robust digital mobility outcomes. These long-term datasets allow for identifying disease trajectories over the disease course on an individual level. (ii) Mobility monitoring in patients’ home environment as demonstrated, for example, in the Mobilise-D project for PD. This real-life mobility data enables to complement established clinical and functional scores by addressing patients’ needs in their daily life. (iii) Technical and clinical validation of additional inertial sensor-based parameters. Filling these gaps will improve the identification of HSP-specific gait patterns, establish relevant gait parameters for clinical trials, and optimize treatment strategies for individuals with HSP.

## Supplementary Information

Supplementary material 1.

## Data Availability

No datasets were generated or analysed during the current study.

## Abbreviations

HSP: Hereditary spastic paraplegia
MOCAP: Motion capture
SPRS: Spastic Paraplegia Rating Scale
EMG: Electromyography
QUADAS-2: Quality Assessment of Diagnostic Accuracy Studies
TEC: Total energy consumption
ROM: Range-of-motion
CP: Cerebral palsy
CA: Cerebellar ataxia
PD: Parkinson’s disease
MAS: Modified Ashworth scale
IMU: Inertial measurement unit
FES: Functional Electrical Stimulation
SPM: Statistical Parametric Mapping
MN: Motor Neuron
A_grf: Area under the ground reaction force curve
CV: Coefficient of variation
FES-I: Falls Efficacy Scale International
GRADE: Grading of Recommendations, Assessments, Development and Evaluation
