# Supplementary material for: Outcome measures of instrumented gait analysis in hereditary spastic paraplegia: a systematic review
Source: J Neuroeng Rehabil. 2025 Jun 5;22:129. doi: 10.1186/s12984-025-01646-4 (PMC12139076; doi:10.1186/s12984-025-01646-4)
Supplement: Supplementary file 1 — Supplementary material 1. [file 12984_2025_1646_MOESM1_ESM.docx]

**Supplementary Information**

Table S1: Search strategy. The figure shows the adapted search strings for each database.

| PubMed | \| # \| Search Query \| \| --- \| --- \| \| 1 \| ("heredi*"[Title/Abstract] AND "spastic parap*"[Title/Abstract]) OR ("heredi*"[Title/Abstract] AND "spastic pare*"[Title/Abstract]) \| \| 2 \| "gait analysis"[MeSH Terms] \| \| 3 \| ("gait"[Title/Abstract] OR "walk*"[Title/Abstract] OR "motion"[Title/Abstract]) \| \| 4 \| ("analys*"[Title/Abstract] OR "asses*"[Title/Abstract] OR "parameter*" [Title/Abstract] OR "measure*" [Title/Abstract] OR "sensor*"[Title/Abstract] OR "feature*" [Title/Abstract] OR "kinematic*" [Title/Abstract] OR "capture" [Title/Abstract] OR "observ*" [Title/Abstract]) \| \| 5 \| #1 AND (#2 OR (#3 AND #4)) \| |
| --- | --- | --- | --- | --- | --- | --- | --- | --- | --- | --- | --- | --- | --- |
| Scopus | (TITLE-ABS-KEY (heredi* AND spastic AND parap*) OR  TITLE-ABS-KEY (heredi* AND spastic AND pare*))  AND  (TITLE-ABS-KEY (gait) OR TITLE-ABS-KEY (walk*) OR TITLE-ABS-KEY (motion))  AND  (TITLE-ABS-KEY (analys*) OR TITLE-ABS-KEY (asses*) OR TITLE-ABS-KEY (parameter*)  OR TITLE-ABS-KEY (measure*) OR TITLE-ABS-KEY (sensor*) OR TITLE-ABS-KEY feature*)  OR TITLE-ABS-KEY ( kinematic*) OR TITLE-ABS-KEY (capture) OR TITLE-ABS-KEY (observ*)) |
| Web of Science | \| # \| Search Query \| \| --- \| --- \| \| 1 \| ((TS=(heredi* AND spastic AND parap*)) OR TS=(heredi* AND spastic AND pare*)) \| \| 2 \| (((TS=(gait)) OR TS=(walk*)) OR TS=(motion)) \| \| 3 \| (TS=(analys*) OR TS=(asses) OR TS=(parameter*) OR TS=(measure*) OR TS=(sensor*)) OR TS=(feature*) OR TS=(kinematic*) OR TS=(capture*) OR TS=(observ*) \| \| 4 \| (#1 and #2 and #3) \| |

Table S2: Details of the risk of bias assessment by study, collected by the QUADAS-2 questionnaire.

| **Author/year** | **Risk of bias** | | | | **Applicability** | | |
| --- | --- | --- | --- | --- | --- | --- | --- |
|  | **Patient selection** | **Index test** | **Reference standard** | **Flow and timing** | **Patient selection** | **Index test** | **Reference standard** |
| van de Venis et al. 2024 | high | high | low | low | low | low | low |
| Ibrahim et al. 2024 | low | low | low | low | low | low | low |
| Beichert et al. 2024 | low | low | low | low | low | low | low |
| Loris et al. 2023 | low | low | low | low | low | low | low |
| van de Venis et al. 2023 | high | low | low | unclear | low | low | low |
| Ollenschläger et al. 2023 | low | high | low | low | low | low | low |
| van Gelder et al. 2023 | low | low | low | high | low | low | low |
| MacWilliams et al. 2022 | unclear | high | high | unclear | high | unclear | unclear |
| Regensburger et al. 2022 | low | low | low | low | low | low | low |
| Joseph et al. 2022 | high | low | high | unclear | low | low | unclear |
| Lassmann et al. 2022 | low | low | low | low | low | low | low |
| van de Venis et al. 2022 | unclear | low | high | unclear | low | low | unclear |
| Coccia et al. 2021 | unclear | low | high | unclear | high | low | unclear |
| Martindale et al. 2020 | low | high | low | low | low | low | low |
| Martiano et al. 2019 | low | high | low | low | low | low | low |
| van Vugt et al. 2019 | high | low | low | low | low | low | low |
| van Lith et al. 2019 | high | low | low | low | low | low | low |
| Pulido-Valdeolivas et al. 2018 | low | low | unclear | low | low | low | low |
| Serrao et al. 2018 | low | low | low | low | high | low | low |
| Martiano et al. 2018 | low | low | low | low | low | high | low |
| Martindale et al. 2018 | low | low | low | low | low | low | low |
| van Lith et al. 2018 | low | low | low | low | low | low | low |
| Rinaldi et al. 2017 | low | high | low | low | low | low | low |
| Martindale et al. 2017 | low | high | low | low | low | low | low |
| Adiar et al. 2016 | unclear | low | high | unclear | low | low | unclear |
| Serrao et al. 2016 | low | high | low | low | low | low | low |
| Riccardo et al. 2016 | low | high | high | low | low | low | low |
| de Niet et al. 2015 | high | low | low | low | low | low | low |
| Zhang et al. 2014 | high | low | low | low | high | low | low |
| Bonnefoy-Mazure et al. 2013 | high | low | unclear | unclear | high | low | low |
| Marsden et al. 2013 | unclear | low | high | unclear | low | low | unclear |
| Mardsen et al. 2012 | unclear | high | high | unclear | low | low | unclear |
| Piccinini et al. 2011 | low | low | low | low | high | low | low |
| Wolf et al. 2011 | low | high | unclear | unclear | high | low | low |
| de Niet et al. 2011 | low | high | low | low | high | low | low |
| Cimolin et al. 2007 | low | low | low | low | high | low | low |
| Klebe et al. 2006 | high | high | low | low | low | low | low |
| Klebe et al. 2004 | high | high | low | low | low | low | low |

Table S 3: Number of parameters in observational studies. Parameters with significant group differences between HSP patients and healthy controls. One study was excluded from the child/adult analysis because it included a mixed cohort of adults and children. In the ‘sig’ columns, the number of parameters is presented that reached significance level.
sig: significant, diffs: differences

|  | **total measured** | **not reported / no controls** | **tested for sig diffs** | **sig** | **child** | | | **adult** | | |
| --- | --- | --- | --- | --- | --- | --- | --- | --- | --- | --- |
|  |  |  |  |  | **total measured** | **tested for sig diffs** | **sig** | **total measured** | **tested for sig diffs** | **sig** |
| **temporal** | 25 | 7 | 18 | 12 | 12 | 1 | 0 | 20 | 18 | 12 |
| **spatial** | 20 | 4 | 16 | 14 | 4 | 3 | 3 | 20 | 16 | 14 |
| **kinematic** | 251 | 137 | 114 | 57 | 214 | 79 | 31 | 44 | 40 | 26 |
| **kinetic** | 25 | 0 | 25 | 13 | 10 | 10 | 8 | 15 | 15 | 5 |
| **EMG** | 11 | 2 | 9 | 7 | 0 | 0 | 0 | 11 | 9 | 7 |
| **other** | 33 | 1 | 32 | 20 | 8 | 7 | 5 | 25 | 25 | 15 |
| **total** | 365 | 151 | 214 | 123 | 248 | 100 | 47 | 135 | 123 | 79 |

EMG: Electromyography, diffs: differences, sig: significant

Table S4: Number of parameters in interventional studies. Significant differences are parameters that significantly differentiate between the treated/untreated group or before/after treatment in interventional studies. For interventional studies, all were tested for significance.

|  | **Outcome measures** | **Significant differences** |
| --- | --- | --- |
| **Temporal** | 10 | 1 |
| **Spatial** | 8 | 5 |
| **kinematic** | 12 | 3 |
| **kinetic** | 10 | 1 |
| **EMG** | 4 | 2 |
| **total** | 44 | 12 |

EMG: Electromyography

Table S5: Spatio-temporal and kinematic parameters significantly correlating with the SPRS (14, 26, 42, 45, 48, 51).

|  | **Parameter correlated to SPRS** |
| --- | --- |
| **temporal** | stride time, stance time and swing time, swing duration %, cadence, double support duration %, MADN double support, CV stride time, CV stance time, CV swing time, CV swing duration |
| **spatial** | walking speed, stride length, SPcmp, cv stride length, lateral step deviation %, circumduction |
| **kinematic** | ROM ankle, ROM knee, ROM shank, ROM foot, ROM thigh, toe ground clearance IC, max heel ground clearance, foot angle at max ground clearance, lower leg angle IC, thigh angle at toe-off, foot angle at toe-off, foot angle at IC, MADN foot angle at IC |

SPRS: Spastic Paraplegia rating scale, CV: coefficient of variation, ROM: range of motion, IC: initial contact, SPcmp: composite measure of spatial step variability, MADN: normalized median absolute deviation


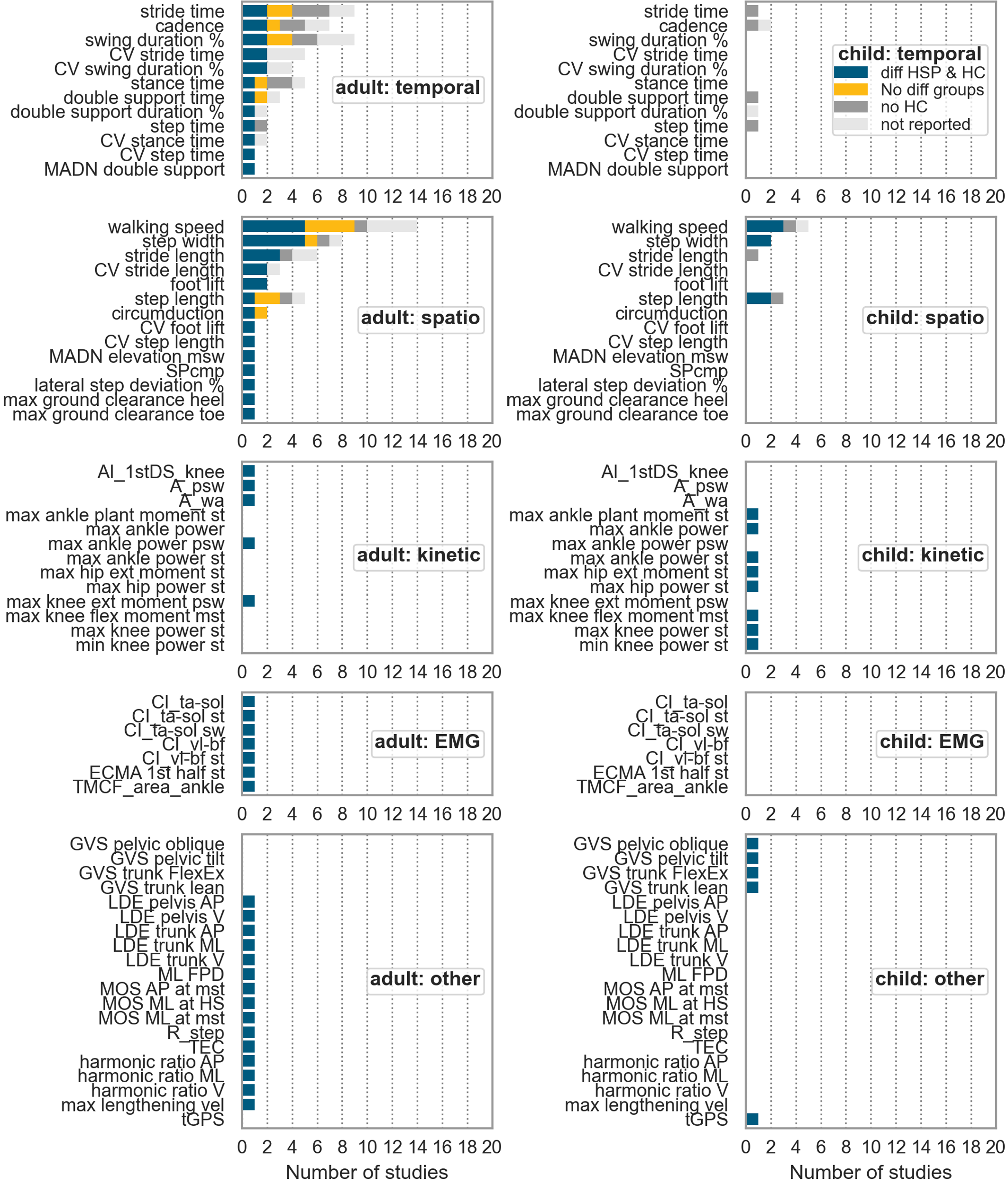


Figure S1: Supplements figure 1: Differences between HSP patients and healthy controls. Adults on the left side and children on the right side All parameters which differentiate between both groups at least in one study were considered. One study was excluded of this analysis, because it included a mixed cohort of adults and children.
nr: Number, diff: difference, HSP: hereditary spastic paraplegia, HC: healthy controls, CV: coefficient of variation, max: maximum, ext: extensor, A:area under the curve, wa: weight acceptance curve, st: stance, mst: mid stance, psw: preswing, AI: angular impulse, DS: double support, CI coactivation index, ta: tibialis-anterior, sol: soleus, TMCf: time-varying multi-muscle co-activation function, sw: swing, vl: vastus lateralis, bf: biceps femoris, ECMA: Early Calf Muscle Activity, R: fraction of mechanical energy, MOS: Margin of Stability, ML: medio-lateral, GVS: Gait Variable Scores, vel: velocity, TEC: total energy consumption, tGPS: Gait profile Score including trunk kinematics, HS: heel strike, FlexEx: flexion/extension, AP: Anteroposterior, ROM: range of motion, IC: initial contact, min: minimum, msw: midswing


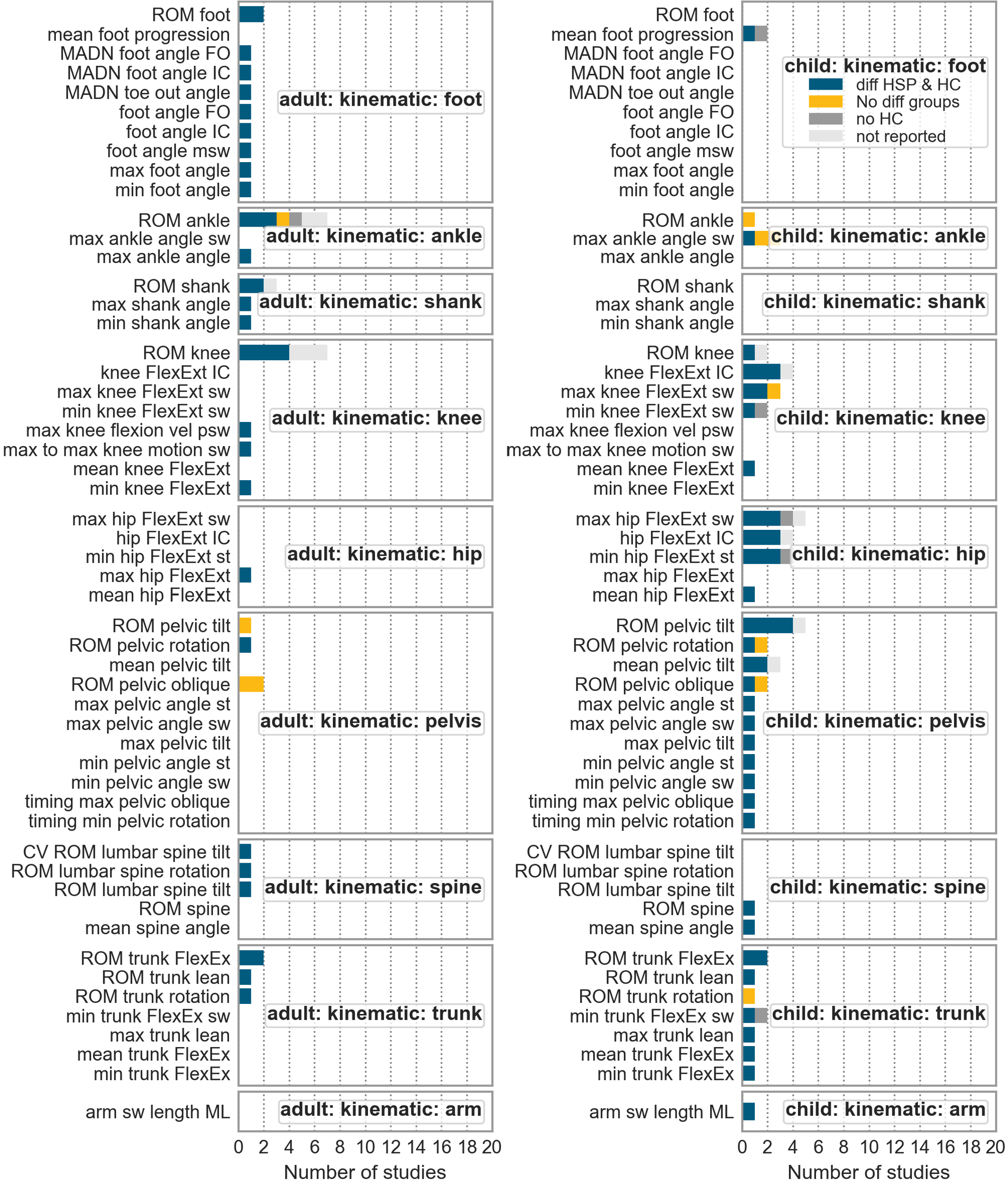


Figure S2: Supplements figure 2: Differences between HSP patients and healthy controls. Adults on the left side and children on the right side. All parameters which differentiate between both groups at least in one study were considered.
.nr: Number, diff: difference, HSP: hereditary spastic paraplegia, HC: healthy controls, ROM: range of motion, CV: coefficient of variation, max: maximum, vel: velocity, HS: heel strike, FlexEx: flexion/extension, AP: Anteroposterior, ROM: range of motion, IC: initial contact, min: minimum, st: stance, msw: midswing
